# Hepatitis B serological markers and plasma DNA concentrations

**DOI:** 10.1097/QAD.0000000000001454

**Published:** 2017-04-25

**Authors:** Huw Price, David Dunn, Tamale Zachary, Tobias Vudriko, Michael Chirara, Cissy Kityo, Paula Munderi, Moira Spyer, James Hakim, Charles Gilks, Pontiano Kaleebu, Deenan Pillay, Richard Gilson

**Affiliations:** University College London, London, UK.

**Keywords:** epidemiology, hepatitis B, hepatitis B ‘e’ antigen, hepatitis B viral load, HIV, Uganda, Zimbabwe

## Abstract

**Objectives:**

To examine hepatitis B (HBV) serological markers and plasma DNA concentrations in a large group of untreated HBV/HIV-coinfected individuals in two sub-Saharan settings.

**Design:**

Baseline analysis of a randomized controlled trial.

**Methods:**

DART was a large trial of treatment monitoring practices in HIV-infected adults with advanced disease starting antiretroviral therapy at centres in Kampala or Entebbe, Uganda (*n* = 2317) and Harare, Zimbabwe (*n* = 999). HBV serological markers [antibody to HBV core antigen, HBV surface antigen (HBsAg), antibody to HBV surface antigen, HBV ‘e’ antigen (HBeAg), and antibody to hepatitis B ‘e’ antigen] and plasma HBV DNA viral load were measured retrospectively on stored baseline samples. Logistic regression was used to examine associations with baseline demographic and clinical factors.

**Results:**

The rate of HBsAg positivity was significantly higher in Zimbabwe than Uganda (12.2 vs. 7.7%, adjusted odds ratio = 1.54, *P* < 0.001) despite a similar prevalence of antibody to HBV core antigen (56.3 vs. 52.4%) in the two settings. Overall, HBsAg positivity was associated with male sex (adjusted odds ratio = 1.54, *P* < 0.001) but not with age, WHO disease stage, or CD4^+^ cell count. HBeAg was detected among 37% of HBsAg-positive patients, with higher rates among those with advanced WHO stage (*P* = 0.02). Also in HBsAg-positive patients, HBV DNA was undetectable in 21%, detectable but below the level of quantification in 14%, and quantifiable in 65%. A total of 96% of HBeAg-positive and 70% of HBeAg-negative patients had detectable HBV DNA; 92 and 28% of patients, respectively, had HBV DNA viral load more than 2000 IU/ml.

**Conclusion:**

High rates of HBV coinfection were observed, highlighting the importance of ensuring that coinfected patients receive an antiretroviral regimen, whether first-line or not, that is active against both viruses.

## Introduction

In the era of antiretroviral treatment (ART), death rates from AIDS-related causes have declined dramatically in both resource-limited and resource-rich regions. In this context, liver disease has emerged as a major cause of death in HIV-infected individuals, although the absolute rates of liver-related mortality have declined [1–3]. Liver-related mortality is increased in those with viral hepatitis coinfection [4].

WHO HIV treatment guidelines state that it is important to determine the local prevalence of hepatitis B (HBV) to inform the decision whether to screen individuals for viral hepatitis, as recommended in resource-rich countries [5]. The use of ART to treat coinfected patients differs from treatment of HIV-monoinfected patients in a number of aspects, in particular ART regimens must have potent activity against both viruses, and treatment interruptions must be avoided because of the potential for liver flares [6–12].

We measured a comprehensive set of HBV serological markers and plasma HBV DNA viral load in archived baseline samples from over 3000 HIV-infected participants in the DART trial. This has allowed a detailed characterization of HBV/HIV coinfection in the regions from where participants were recruited, namely, Kampala/Entebbe, Uganda and Harare, Zimbabwe.

## Methods

DART was a randomized open-label trial of monitoring practices in HIV-infected adult patients starting antiretroviral therapy, conducted at clinical centres in Uganda [Medical Research Council/Uganda Virus Research Institute (UVRI) Uganda Research Unit on AIDS, Entebbe (25 mi from Kampala); Joint Clinical Research Centre (JCRC), Kampala; and satellite Infectious Diseases Institute, Mulago, Kampala] and Zimbabwe (University of Zimbabwe, Harare). Patients were randomized to clinically driven monitoring only (CDM) or clinical monitoring and routine laboratory monitoring in the form of 12-weekly CD4^+^ and haematological/biochemical toxicity tests. Results were not returned for patients in the CDM arm unless specifically requested by the patient's doctor or if a grade 4 toxicity occurred. All participants started first-line ART with zidovudine and lamivudine and either tenofovir, nevirapine, or abacavir. Inclusion criteria were: age at least 18 years, CD4^+^ cell count less than 200 cells/μl, and naive to ART except for exposure for the prevention of mother-to-child transmission. Exclusion criteria were: likely to be unable to attend follow-up, likely to have poor compliance, acute infection including intense phase of tuberculosis treatment, malignancy requiring chemotherapy, laboratory test result indicative of contraindication to ART (including alanine transaminase greater than five times the upper limit of normal), pregnancy, and breastfeeding [13].

**Fig. 1:**
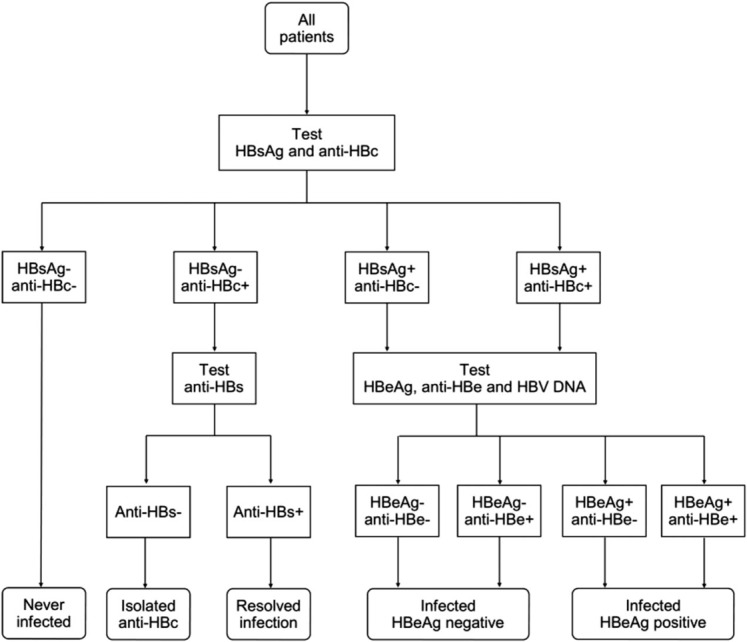
Algorithm for hepatitis B serology testing.

Plasma samples were stored at screening, enrolment, and each scheduled 3-monthly clinic visit. The current study describes the results of serological and virological tests for HBV that were performed retrospectively on the screening or enrolment sample for all participants, according to the algorithm in Fig. 1. All patients were tested for HBV surface antigen (HBsAg) and antibody to HBV core antigen (anti-HBc). Those with detectable anti-HBc without HBsAg were tested for antibody to HBV surface antigen (anti-HBs). Those with detectable HBsAg were tested for HBeAg, antibody to hepatitis B ‘e’ antigen (anti-HBe), and HBV DNA.

anti-HBc, antibody to hepatitis B core antigen; anti-HBe, antibody to hepatitis B ‘e’ antigen; anti-HBs, antibody to hepatitis B surface antigen; HBeAg, hepatitis B ‘e’ antigen; HBsAg, hepatitis B surface antigen; HBV DNA; hepatitis B DNA viral load.

Serological and HBV DNA viral load assays were conducted locally, except for the testing of HBeAg, anti-HBe, and HBV DNA on UVRI samples, which were transported to and tested at JCRC. All sites used commercial serological assays [UVRI and Harare: Murex (Diasorin, Saluggia, Piedmont, Italy), based on an enzyme immunoassay method; JCRC: Roche Elecsys, an electrochemoluminescence assay (Roche Diagnostics Limited, Rotkreuz, Zug, Switzerland)]. The HBsAg assays were known to be unaffected by recognized HBsAg mutants; positive results were confirmed by a neutralization test. Anti-HBs results were classified as positive if the concentration was more than 10 mIU/ml. For the quantification of HBV DNA, JCRC used Roche Cobas Ampliprep/Cobas TaqMan (lower limit of detection 12 IU/ml, upper limit of quantification 110 × 10^6^ IU/ml), whereas Harare used Abbott RealTi*m*e HBV after manually preparing samples using the *m*Sample Preparation System_DNA_ (lower limit of detection 10 IU/ml, upper limit of quantification 1 × 10^9^ IU/ml). Due to low sample volumes, all samples at JCRC were diluted 1 : 4, giving a quantitative range of 48–440 × 10^6^ IU/ml. Both JCRC and Harare participated in the United Kingdom National External Quality Assessment Service (UKNEQAS) scheme. Results from the three Uganda sites were broadly similar and have been combined in the analysis.

### Statistical methods

Multivariable logistic regression was used to examine associations between baseline factors and anti-HBc, HBsAg, HBeAg status, and HBV DNA viral load concentration (dichotomized using a cut-off of 2000 IU/ml [14,15]). *P* values for continuous and ordered variables (age, CD4^+^, and WHO stage) are presented from models fitting each variable as a continuous factor. Pairwise interactions between baseline factors (all possible combinations) were assessed by adding these to the model individually in addition to the main effects; in view of the large number of interaction terms examined, only those significant at *P* value less than 0.01 are reported.

## Results

Selected baseline characteristics of the DART population are shown in Table 1 and have previously been reported in more detail [13]. There were 2317 participants from Uganda and 999 from Zimbabwe. Median age was 36 years, and women outnumbered men (ratio 1.9 : 1, *P* < 0.001). Reflecting the inclusion criteria, the population had advanced infection; median CD4^+^ cell count was 86 cells/μl, and 23% had previously been diagnosed with a WHO stage 4 illness.

### Serological findings

Data completeness was excellent, with only five (0.2%) participants not tested for anti-HBc and one (0.04%) not tested for HBsAg. The rate of anti-HBc positivity was similar in participants from the sites in Uganda (52%) and Zimbabwe (56%). Men were significantly more likely to test positive than women [adjusted odds ratio (aOR) 1.39, 95% confidence interval (CI) 1.19–1.61], although this difference was more marked in Zimbabwe (aOR 1.85, 95% CI 1.41–2.42) than Uganda (aOR 1.22, 95% CI 1.02–1.46) (*P* = 0.005, test for interaction). No association was observed with WHO stage or baseline CD4^+^ cell count.

A total of 308 (9%) patients were found to be HBsAg-positive, 54 (18%) of whom were anti-HBc-negative, although most (*n* = 31) of them had other evidence of HBV infection in the form of detectable HBeAg, anti-HBe, or HBV DNA. A significantly higher rate (*P* = 0.001) of anti-HBc-negativity was observed in Zimbabwe (40/167; 24%) than in Uganda (14/140; 10%), and evidence of a higher rate at lower ages (*P* = 0.04, test for trend). No associations were observed for the other variables examined (sex, WHO stage, and CD4^+^ cell count).

In contrast to the similar rates of anti-HBc positivity, HBsAg was detected much more frequently (aOR 2.99, 95% CI 2.35–3.81) in Zimbabwean patients (17%) than in Ugandan patients (6%). HBsAg positivity was significantly higher in men (aOR 1.54, 95% CI 1.20–1.97) but there was no association with age, WHO stage, or CD4^+^ cell count. Of 1505 patients who were anti-HBc-positive and HBsAg-negative, 962 (64%) were anti-HBs-positive, consistent with a resolved infection and natural immunity.

A total of 280 (91%) patients who were HBsAg-positive had sufficient sample to allow further testing for HBeAg and anti-HBe. A total of 103 (37%) were HBeAg-positive and 127 (45%) were anti-HBe-positive; six (2%) patients had dual positive results and 56 (20%) dual negative results. HBeAg positivity was not associated with country or age, although there was a trend towards a higher prevalence in men (44%) than in women (31%) (*P* = 0.10) (Table 2). Rates of HBeAg positivity were higher in patients with more advanced HIV infection, as reflected by WHO stage (*P* = 0.02) and CD4^+^ cell count (*P* = 0.09).

Further details of serological results are shown in Appendix 1.

### Hepatitis B DNA viral load

Of 308 patients with a positive HBsAg result, 270 (88%) with available samples were tested for HBV DNA viral load. A total of 56 (21%) had undetectable DNA, 38 (14%) had DNA detectable but below the level of quantification, and 176 (65%) had a quantifiable level of DNA. The detection of HBV DNA viral load was strongly linked to HBeAg status, 96% (80/83) of HBeAg-positive and 70% (117/167) of HBeAg-negative participants having detectable levels (*P* < 0.001).

**Fig. 2:**
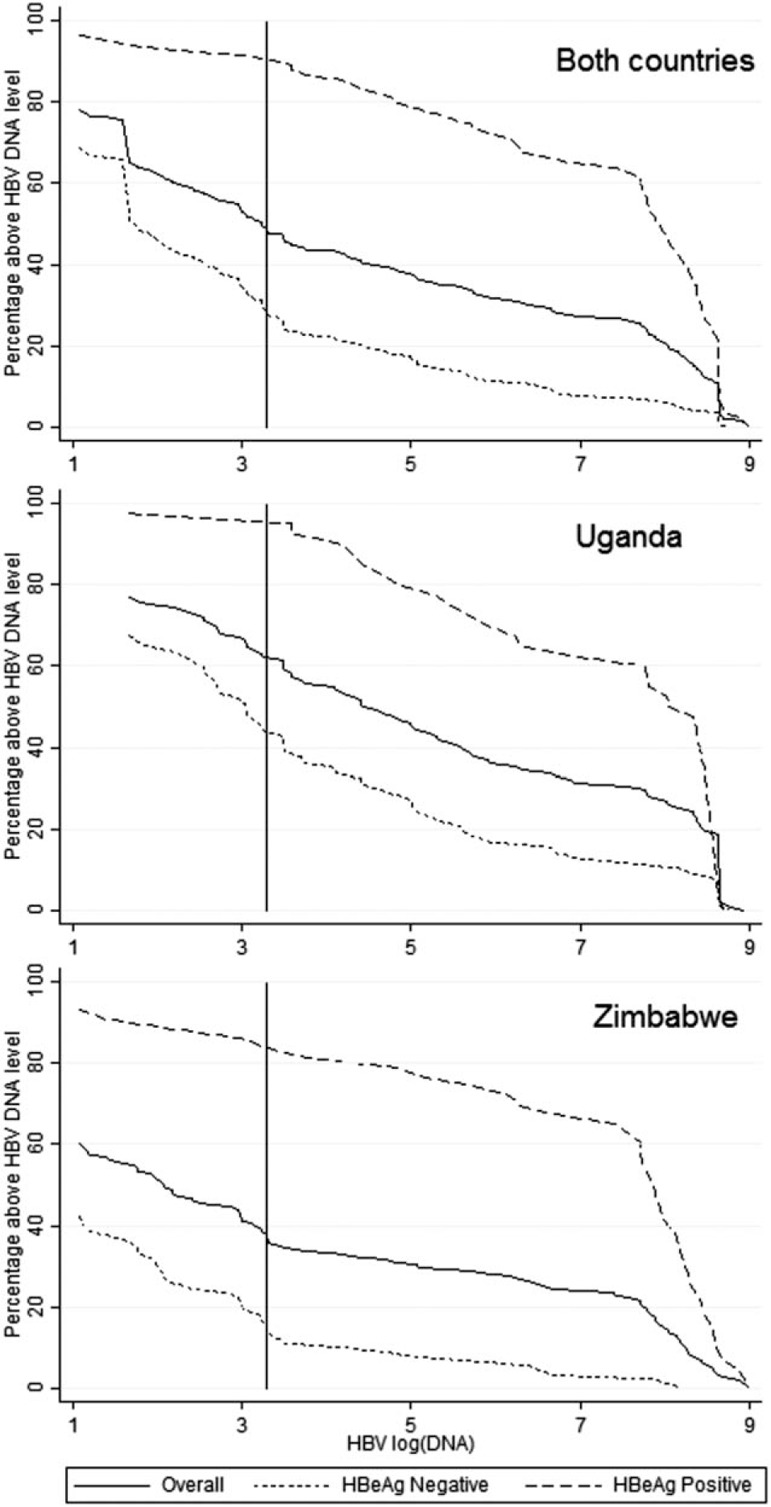
Hepatitis B DNA viral load by hepatitis B ‘e’ antigen status.

Figure 2 shows the distribution of HBV DNA viral loads, by country and HBeAg status, in terms of the percentage of samples that exceed a given concentration. A vertical line is drawn at 2000 IU/ml, the threshold for initiating anti-HBV treatment according to some guidelines [14,15]. Overall, 92% of HBeAg-positive and 28% of HBeAg-negative participants had HBV DNA viral load more than 2000 IU/ml. As expected, HBV DNA levels were generally high among HBeAg-positive patients irrespective of clinical site, 53% having a value greater than 1 × 10^8^ IU/ml. Values were spread more uniformly among HBeAg-negative patients and appeared on average to be lower in Zimbabwean than in Ugandan patients.

Table 3 shows the results of a multivariable logistic regression model predicting an outcome of HBV DNA viral load more than 2000 IU/ml. Models were fitted with and without a term for HBeAg status; these have different interpretations as both parameters are essentially measuring HBV replication. As suggested by Fig. 2, Zimbabwean patients were significantly less likely (*P* < 0.001) to have an HBV DNA level above this threshold, largely driven by differences among HBeAg-negative patients. The only other significant factor was WHO stage (in the model that did not adjust for HBeAg status), with a higher probability of having HBV DNA viral load more than 2000 IU/ml the more advanced the stage (*P* = 0.03).

## Discussion

The description of the seroepidemiology of hepatitis B in HIV-infected adults in sub-Saharan Africa is largely limited to HBsAg and anti-HBc. In a systematic review of these markers, Barth *et al.*[16] reported an average HBsAg prevalence of 15%, but with a very wide range from 4 to 70%, and with variation occurring both between and within countries. The 6% HBsAg positivity rate found in DART participants from Kampala/Entebbe is somewhat lower than estimates from previous studies in this region of Uganda; the 17% rate in participants from Harare is somewhat higher than previous studies [17–25]. Notably, the overall prevalence of anti-HBc, with just over one-half of participants having evidence of exposure to the virus, was similar in the two countries. As vertical transmission or infection in the first few years of life is the strongest determinant of developing chronic infection, this suggests that the proportion infected early in life is higher in Zimbabwe than in Uganda. We found a slight increase in the prevalence of anti-HBc with increasing age, which may indicate continuing infection during adulthood but may also be a cohort effect, with historically declining transmission. HBsAg was detectable despite undetectable anti-HBc in 54 (1.6%) study participants. The higher rate in Zimbabwe could be due to biological differences between the populations or the use of different serological assays. The prevalence of this atypical pattern has been described to range between 4 and 56% of those with detectable HBsAg [17,26], and in differing situations including in neonates, in immunosuppression, and in the presence of core gene mutations [26–29]. In HIV-positive individuals, it is associated with a low CD4^+^ cell count, sometimes with development of an anti-HBc response on starting ART [30]. As an anti-HBc test is sometimes used to screen patients prior to an HBsAg test, this testing strategy may fail to identify some HBsAg-positive patients [31].

A total of 543 participants, 30.0% of those with evidence of HBV exposure, had isolated anti-HBc. Similar rates (32–42%) have been found in previous studies in Uganda and elsewhere in sub-Saharan Africa [32–35]. This pattern may be due to false-positive anti-HBc test results, rare in a high prevalence population such as this, or be transient and occur during the resolution phase of acute HBV. Persistent isolated anti-HBc may also be due to occult HBV infection (with low-level detectable HBV DNA viral load) or loss of anti-HBs with time or immunosuppression in patients who have resolved infection. Repeat serology and HBV DNA viral load testing would help to determine more accurately the status of the 543 patients with isolated anti-HBc, but was not available in this study.

The major novel contribution from our study in an HIV-positive population in Africa is extensive data on HBeAg and HBV DNA viral load, the most powerful prognostic markers for disease progression and viral transmission. Previous studies are either based on small sample sizes or do not distinguish HIV-uninfected and HBV/HIV-coinfected individuals. A previous study of mostly HIV-negative, HBsAg-seropositive inpatients in Kampala found 27% HBeAg seropositive [22]. An earlier study of inpatients in the same hospital found six (28.1%) of 23 HIV-positive and three (17.6%) of 17 HIV-negative patients to be HBeAg seropositive [18]. A small study of HIV-infected pregnant women in Uganda and Rwanda found that three (33%) of nine with detectable HBsAg were HBeAg seropositive [19]. In Zimbabwe, rates of HBeAg seropositivity ranged from 3.3% in pregnant women in Harare [36] to 24.5% [37] in a national survey, but neither study tested for HIV. In HIV/HBV-coinfected Zimbabwean patients recruited to a randomized controlled trial, 54.2% (13 of 24) were HBeAg seropositive [25]. In the DART population, we found that approximately one-third of HBsAg-positive patients were HBeAg positive. As expected, HBeAg status was intimately linked to HBV DNA viral load, with very high levels observed in HBeAg-positive patients. Nonetheless, 28% of HBeAg-negative patients had a viral load greater than 2000 IU/ml, the threshold for considering anti-HBV treatment in guidelines [14,15]. In the small study of HBV/HIV-coinfected pregnant women in Uganda and Rwanda, three of five HBeAg-negative patients had detectable HBV DNA with a mean viral load of 1700 IU/ml [19]. In the randomized controlled trial cited above [25], the 24 Zimbabwean patients were included in a larger cohort (*n* = 115) in which at least 28% of HBeAg-negative participants had HBV DNA greater than 2000 IU/ml [10]. Among HBeAg-positive patients, HBV DNA was detected more frequently, and the distribution of viral load values was higher in Uganda than in Zimbabwe. The use of different viral load assays is one possible explanation for this finding, although both laboratories participated in the same external quality control scheme.

Immunosuppression associated with HIV coinfection can result in reactivation of HBV infection, with reappearance of HBsAg or HBeAg, or a reduction in the rate of loss of either marker over time. Without an HIV-negative comparator population, we were not able to examine this; however, we were able to assess the influence of the degree of immunosuppression as measured by WHO clinical stage and CD4^+^ cell count at study entry. No clear associations were observed for HBsAg status, but HBeAg positivity was markedly higher in those with a more advanced WHO stage of disease, and there was a consistent, albeit nonsignificant, trend with CD4^+^ cell count. The effect of WHO stage was mirrored in an analysis of the proportion of participants with HBV DNA levels greater than 2000 IU/ml. Reactivation of HBV infection could explain the higher liver-related mortality that has been observed in HBV/HIV-coinfected individuals [4]. An association between advanced disease (in this case low CD4^+^ cell count) and both HBeAg and HBsAg status was noted previously in a study in Nigeria, although these investigators suggested causation to be acting in the other direction, namely, active HBV infection lowering CD4^+^ cell count [38].

The prevalence of anti-HBc was significantly higher in male participants than in female participants, particularly in Zimbabwe. An even more pronounced sex difference was observed for HBsAg, consistent with other studies that have shown that men are less likely to clear HBV and progress to chronic infection, either when infected in childhood or as adults [39–42]. There was also a nonsignificant trend of a higher rate of HBeAg positivity among male participants, although no evidence of a sex difference in HBV DNA concentrations exists.

The strengths of our study are the large sample size, the very high rate of sample retrieval (close to 100%), detailed clinical and demographic data, the comprehensive range of virological markers tested, and that all the laboratories were participating in the UKNEQAS quality assurance programme. The main limitation is the testing of participants at a single time point, precluding the estimation of HBV incidence and the ability to determine with certainty that all the HBsAg positivity was due to chronic HBV infection or whether there may have been some acute infections that may resolve. It is unlikely that acute infection contributes substantially to HBsAg prevalence as most transmission in sub-Saharan Africa occurs in childhood. Another limitation is that we did not perform HBV DNA assays on the surprisingly large number of patients (*n* = 543) who had isolated anti-HBc. This serological pattern may represent a false-positive, resolved and cleared infection, or chronic infection with a low rate of viral replication (occult HBV infection). In a previous small study in Uganda, 15% (seven of 48) HIV-positive patients with negative HBsAg had detectable HBV DNA [22]. The clinical implications of occult HBV infection are unclear, but it is generally accepted that individuals with detectable plasma HBV DNA may be at risk of HBV reactivation and inflammatory liver flares [43]. Finally, DART was limited to patients with a CD4^+^ cell count less than 200 cells/μl and thus did not include those with less-advanced HIV infection. This may have limited our ability to identify associations with this key marker of immunosuppression.

In conclusion, high rates of active HBV infection were observed in both geographical settings in Africa, highlighting the importance of considering HBV coinfection in patients receiving antiretroviral drugs, regardless of whether this is their first or subsequent regimen, and using agents that are active against both viruses. Further analyses of longitudinal data in DART are ongoing, including the impact of chronic HBV infection on mortality and whether coinfected patients who received lamivudine without tenofovir had less favourable virological outcomes compared with those who received both drugs.

## Acknowledgements

We thank all the patients and staff from all the centres participating in the DART trial.

DART was funded by the UK Medical Research Council (grant number G0600344), the UK Department for International Development (DFID), and the Rockefeller Foundation. GlaxoSmithKline/ViiV Healthcare, Gilead, Boehringer Ingelheim, and AbbVie Inc. donated drugs for DART. Gilead Sciences funded the HBV immunological and virological assays. H.P. was funded by a UK Medical Research Council Clinical Research Training Fellowship.

Authors’ contributions: H.P. – designed and coordinated study, carried out analysis, wrote first draft, and revised subsequent versions; D.D. – designed and coordinated study, advised on analysis, cowrote first draft, and revised subsequent versions; T.Z. and T.V. – carried out laboratory testing, commented on drafts; M.C. – carried out laboratory testing; C.K., P.M., and J.H. – site PI for main trial, commented on drafts; M.S. – coordinated study, commented on drafts; C.G. and D.P. – designed study, commented on drafts; P.K. – commented on drafts; R.G. – designed and coordinated study, commented on drafts.

Justification of the number of contributors greater than 10: DART was a very large international study with a large study group.

**DART Virology Group**: P. Kaleebu (Co-Chair), D. Pillay (Co-Chair), P. Awio, M. Chirara∗, D. Dunn, D.M. Gibb, C. Gilks, R. Goodall, A. Kapaata, M. Katuramur, F. Lyagoba, B. Magambo, K. Mataruka, L. Mugarura, T. Musunga, M. Nabankkema, J. Nkalubo, P. Nkurunziza, C. Parry, V. Robertson, M. Spyer, D. Mulima, D.E. Williams, I. Nankya, S. Nassimbwa, E. Ndashimye, E. Nabulime, M. Phiri, K. Mutasa, and S. Mukasa. **MRC/UVRI Uganda Research Unit on AIDS, Entebbe, Uganda:** H. Grosskurth, P. Munderi, G. Kabuye, D. Nsibambi, R. Kasirye, E. Zalwango, M. Nakazibwe, B. Kikaire, G. Nassuna, R. Massa, K. Fadhiru, M. Namyalo, A. Zalwango, L. Generous, P. Khauka, N. Rutikarayo, W. Nakahima, A. Mugisha, J. Todd, J. Levin, S. Muyingo, A. Ruberantwari, P. Kaleebu, D. Yirrell, N. Ndembi, F. Lyagoba, P. Hughes, M. Aber, A. Medina Lara, S. Foster, J. Amurwon, and B. Nyanzi Wakholi.

**Joint Clinical Research Centre, Kampala, Uganda:** P. Mugyenyi, C. Kityo, F. Ssali, D. Tumukunde, T. Otim, J. Kabanda, H. Musana, J. Akao, H. Kyomugisha, A. Byamukama, J. Sabiiti, J. Komugyena, P. Wavamunno, S. Mukiibi, A. Drasiku, R. Byaruhanga, O. Labeja, P. Katundu, S. Tugume, P. Awio, A. Namazzi, G.T. Bakeinyaga, H. Katabira, D. Abaine, J. Tukamushaba, W. Anywar, W. Ojiambo, E. Angweng, S. Murungi, W. Haguma, S. Atwiine, and J. Kigozi.

**University of Zimbabwe, Harare, Zimbabwe:** A. Latif, J. Hakim, V. Robertson, A. Reid, E. Chidziva, R. Bulaya-Tembo, G. Musoro, F. Taziwa, C. Chimbetete, L. Chakonza, A. Mawora, C. Muvirimi, G. Tinago, P. Svovanapasis, M. Simango, O. Chirema, J. Machingura, S. Mutsai, M. Phiri, T. Bafana, M. Chirara, L. Muchabaiwa, and M. Muzambi.

**Infectious Diseases Institute (formerly the Academic Alliance) Makerere University, Mulago, Uganda:** E. Katabira, A. Ronald, A. Kambungu, F. Lutwama, A. Nanfuka, J. Walusimbi, E. Nabankema, R. Nalumenya, T. Namuli, R. Kulume, I. Namata, L. Nyachwo, A. Florence, A. Kusiima, E. Lubwama, R. Nairuba, F. Oketta, E. Buluma, R. Waita, H. Ojiambo, F. Sadik, J. Wanyama, and P. Nabongo.

**The AIDS Support Organisation (TASO), Uganda:** R. Ochai and D. Muhweezi.

**Imperial College, London, UK:** C. Gilks, K. Boocock, C. Puddephatt, D. Winogron, and J. Bohannon.

**MRC Clinical Trials Unit at UCL, London, UK:** J. Darbyshire, D.M. Gibb, A. Burke, D. Bray, A. Babiker, A.S. Walker, H. Wilkes, M. Rauchenberger, S. Sheehan, L. Peto, K. Taylor, M. Spyer, A. Ferrier, B. Naidoo, D. Dunn, and R. Goodall.

**Independent DART Trial Monitors:** R. Nanfuka and C. Mufuka-Kapuya.

**Trial Steering Committee:** I. Weller (Chair), A. Babiker (Trial Statistician), S. Bahendeka, M. Bassett, A. Chogo Wapakhabulo, J. Darbyshire, B. Gazzard, C. Gilks, H. Grosskurth, J. Hakim, A. Latif, C. Mapuchere, O. Mugurungi, P. Mugyenyi; Observers: C. Burke, M. Distel, S. Jones, E. Loeliger, P. Naidoo, C. Newland, G. Pearce, S. Rahim, J. Rooney, M. Smith, W. Snowden, J.-M. Steens, and M. Ait-Khaled.

**Data and Safety Monitoring Committee:** A. Breckenridge (Chair), A. McLaren (Chair-deceased), C. Hill, J. Matenga, A. Pozniak, and D. Serwadda.

**Endpoint Review Committee**: T. Peto (Chair), A. Palfreeman, M. Borok, and E. Katabira.

### Conflicts of interest

There are no conflicts of interest.

## Supplementary Material

Supplemental Digital Content

## Figures and Tables

**Table 1:** Baseline predictors of antibody to HBV core antigen-positivity and hepatitis B surface antigen-positivity: logistic regression analysis.

	Total	Anti-HBc positivity					HBsAg positivity				
	*N*	*n*/*N*	%	aOR	95% CI	*P*	*n*/*N*	%	aOR	95% CI	*P*
All	3316	1774/3311	53.6				308/3315	9.3			
Site						0.16					<0.001
Uganda	2317	1214/2316	52.4	1.00			141/2317	6.1	1.00		
Zimbabwe	999	560/995	56.3	1.12	0.96–1.30		167/998	16.7	2.99	2.35–3.81	
Sex						<0.001					<0.01
Female	2156	1083/2152	50.3	1.00			167/2155	7.7	1.00		
Male	1160	691/1159	59.6	1.39	1.19–1.61		141/1180	12.2	1.54	1.20–1.97	
Age (years)						<0.001					0.16
18–29	532	249/531	46.9	1.00			46/532	8.6	1.00		
30–34	796	407/794	51.3	1.15	0.92–1.43		79/795	9.9	1.06	0.72–1.56	
35–39	848	454/848	53.5	1.21	0.97–1.51		77/848	9.1	0.89	0.60–1.32	
40–44	608	349/607	57.5	1.40	1.11–1.78		63/608	10.4	1.01	0.67–1.52	
45–49	313	180/312	57.7	1.40	1.05–1.86		31/313	9.9	0.93	0.57–1.52	
50 and over	219	135/219	61.6	1.63	1.18–2.26		12/219	5.5	0.49	0.25–0.95	
WHO stage						0.88					0.84
2	673	364/673	54.1	1.00			61/672	9.1	1.00		
3	1864	1002/1861	53.8	0.99	0.83–1.18		178/1864	9.5	1.01	0.74–1.38	
4	779	408/777	52.5	0.98	0.79–1.22		69/779	8.9	1.04	0.71–1.51	
CD4^+^ cell count (cells/μl)						0.27					0.97
<50	1109	576/1107	52.0	1.00			99/1109	8.9	1.00		
50–99	785	431/783	55.0	1.10	0.91–1.33		84/784	10.7	1.21	0.88–1.65	
100–149	759	403/759	53.1	1.06	0.87–1.28		68/759	9.0	1.05	0.75–1.47	
150–199	663	364/662	55.0	1.14	0.93–1.39		57/663	8.6	1.01	0.71–1.44	

All odds ratio and *P* values are from a multivariable model including all covariates shown. Statistical significance of age, WHO stage, and CD4^+^ cell count assessed by test for trend. There were no significant (*P* < 0.01) two-way interactions except site and sex (see text). Anti-HBc, antibody to hepatitis B core antigen; aOR, adjusted odds ratio; CI, confidence interval; HBsAg, hepatitis B surface antigen.

**Table 2:** Baseline predictors of hepatitis B ‘e’ antigen-positivity in those testing hepatitis B surface antigen positive: logistic regression analysis.

	HBeAg positivity				
	Positive				
	*n*/*N*	%	aOR	95% CI	*P*
All	103/280	36.8			
Site					0.33
Uganda	51/130	39.2	1.00		
Zimbabwe	52/150	34.7	0.78	0.46–1.30	
Sex					0.10
Female	48/155	31.0	1.00		
Male	55/125	44.0	1.56	0.92–2.63	
Age (years)					0.71
18–34	36/111	32.4	1.00		
35–39	25/67	37.3	1.16	0.60–2.25	
40–44	27/61	44.3	1.50	0.76–2.98	
>45	15/41	36.6	1.21	0.54–2.70	
WHO stage					0.02
2	14/59	23.7	1.00		
3	57/159	35.8	1.79	0.88–3.65	
4	32/62	51.6	3.28	1.44–7.48	
CD4^+^ cell count (cells/μl)					0.09
<50	36/89	40.0	1.00		
50–99	35/74	47.3	1.49	0.78–2.86	
100–149	16/65	24.6	0.57	0.27–1.19	
150–199	16/52	30.8	0.87	0.40–1.91	

Note that fewer age groups used than in Table 1 to avoid small numbers. All odds ratio and *P* values are from a multivariable model including all covariates shown. Statistical significance of age, WHO stage, and CD4^+^ cell count assessed by test for trend. There were no significant (*P* < 0.01) two-way interactions. aOR, adjusted odds ratio; CI, confidence interval; HBeAg, hepatitis B ‘e’ antigen.

**Table 3:** Baseline predictors of hepatitis B DNA more than 2000 IU/ml: logistic regression analysis.

		>2000 IU/ml		Not adjusted for HBeAg			Adjusted for HBeAg		
		*n*/*N*	%	aOR	95% CI	*P*	aOR	95% CI	*P*
All		131/270	49						
Site	Uganda	76/122	62	1.00		<0.001	1.00		<0.001
	Zimbabwe	55/148	37	0.32	0.19–0.54		0.20	0.10–0.40	
Sex	Male	60/118	51	1.00		0.65	1.00		0.76
	Female	71/152	47	0.88	0.52–1.51		1.11	0.57–2.15	
Age group (years)	18–34	54/114	47	1.00		0.88	1.00		0.29
	35–39	30/61	49	1.04	0.53–2.03		1.02	0.46–2.27	
	40–44	31/55	56	1.43	0.71–2.87		1.17	0.49–2.82	
	45 and over	16/40	40	0.72	0.33–1.61		0.42	0.14–2.82	
WHO stage	2	20/56	36	1.00		0.03	1.00		0.59
	3	76/154	49	1.95	0.98–3.89		1.71	0.73–4.05	
	4	35/60	58	2.60	1.14–5.95		1.35	0.47–3.84	
Baseline CD4^+^ cell count (cells/μl)	<50	43/87	49	1.00		0.57	1.00		0.55
	50–99	42/73	58	1.47	0.76–2.87		1.09	0.48–2.46	
	100–149	20/58	34	0.52	0.24–1.10		0.48	0.19–1.20	
	150–199	26/52	50	1.12	0.51–2.44		0.90	0.35–2.32	

Analysis based on hepatitis B surface antigen-positive patients. Note that fewer age groups used than in Table 1 to avoid small numbers. All odds ratio and *P* values are from a multivariable model including all covariates shown. Statistical significance of age, WHO stage, and CD4^+^ cell count assessed by test for trend. There were no significant (*P* < 0.01) two-way interactions. aOR, adjusted odds ratio; CI, confidence interval; HBeAg, hepatitis B ‘e’ antigen.
