# Supplementary material for: Hepatitis B serological markers and plasma DNA concentrations
Source: AIDS. 2017 Apr 25;31(8):1109–17. doi: 10.1097/QAD.0000000000001454 (PMC5414544; doi:10.1097/QAD.0000000000001454)
Supplement: Supplemental Digital Content [file aids-31-1109-s001.docx]

Appendix 1 – combinations of All serology results

| **Anti-HBc** | **HBsAg** | **Anti-HBs** | **HBeAg** | **Anti-HBe** | **Frequency** |
| --- | --- | --- | --- | --- | --- |
|  | NEG | NEG |  |  | 3 |
|  | NEG | POS |  |  | 1 |
|  | POS |  | NEG | POS | 1 |
| NEG |  | NEG |  |  | 1 |
| NEG | NEG |  |  |  | 1166 |
| NEG | NEG |  | NEG | NEG | 12 |
| NEG | NEG |  | NEG | POS | 1 |
| NEG | NEG | NEG |  |  | 270 |
| NEG | NEG | NEG | NEG | NEG | 6 |
| NEG | NEG | POS |  |  | 26 |
| NEG | NEG | POS | NEG | NEG | 1 |
| NEG | POS |  |  |  | 5 |
| NEG | POS |  | NEG | NEG | 25 |
| NEG | POS |  | NEG | POS | 2 |
| NEG | POS |  | POS | NEG | 15 |
| NEG | POS |  | POS | POS | 1 |
| NEG | POS | NEG | NEG | NEG | 3 |
| NEG | POS | NEG | NEG | POS | 1 |
| NEG | POS | POS |  |  | 1 |
| NEG | POS | POS | NEG | NEG | 1 |
| POS | NEG |  |  |  | 13 |
| POS | NEG |  | NEG | POS | 3 |
| POS | NEG | NEG |  |  | 533 |
| POS | NEG | NEG | NEG | POS | 6 |
| POS | NEG | NEG | POS | NEG | 3 |
| POS | NEG | NEG | POS | POS | 1 |
| POS | NEG | POS |  |  | 950 |
| POS | NEG | POS | NEG | NEG | 5 |
| POS | NEG | POS | NEG | POS | 7 |
| POS | POS |  |  |  | 19 |
| POS | POS |  | NEG | NEG | 19 |
| POS | POS |  | NEG | POS | 94 |
| POS | POS |  | POS | NEG | 71 |
| POS | POS |  | POS | POS | 4 |
| POS | POS | NEG |  |  | 1 |
| POS | POS | NEG | NEG | NEG | 4 |
| POS | POS | NEG | NEG | POS | 18 |
| POS | POS | NEG | POS | NEG | 10 |
| POS | POS | NEG | POS | POS | 1 |
| POS | POS | POS |  |  | 2 |
| POS | POS | POS | NEG | NEG | 4 |
| POS | POS | POS | NEG | POS | 5 |
| POS | POS | POS | POS | NEG | 1 |
| **Total** |  |  |  |  | **3316** |
